# Evaluation of health determinants for sustaining workability in aging US workforce

**DOI:** 10.4236/aar.2013.23015

**Published:** 2013-08

**Authors:** Vatsalya Vatsalya, Robert Karch

**Affiliations:** Department of Health Promotion and Management, College of Arts and Sciences, American University, Washington DC, USA

**Keywords:** Assessment, Health, Older, Sustainability, Workability, Work-Life

## Abstract

Growth of older population in United States requires multi-generational evaluation to characterize health measures for sustaining workability. Investigation of measures that working population would need and use with their work-life in an attempt to stay healthy and fit, could potentially reveal significant association that could extend workability and enhance work productivity such as performance, presenteeism, job satisfaction. Evaluation with selective longitudinal health profiling; employment prerequisites; socio-economic and psychological scales could characterize health measures significantly associated with work sustainability. Such health measures could potentially be employed by US working population early in their life and occupation to sustain and improve workability in their later epoch.

## 1. PRESENT GLOBAL HEALTH CONCERNS AND TRENDS OF WORK-LIFE EXPECTANCY

Rising health concerns and financial deficits have been adversely influencing economies of many nations and population worldwide in the last decade. Globally, countries are trying extensively to intervene with their financial crisis and there by over-burdening citizens with various economic reforms and taxations [1]. Ramifications of workability have dramatically evolved as well, and employees are growingly anticipated to perform better both qualitatively and quantitatively more than ever with early retirement and job-loss becoming common in working set-ups. At the same time, relative age-group of the population worldwide has been tending largely towards an older faction with increased longevity supported from the advancements in healthcare and its availability. Older population also need accessibility of robust healthcare services for appropriate geriatric ailments, which have sharply become expensive in the last few decades with the rise in health-risk behavior and disability incidents [2]. Variable working paradigms, lifestyle management towards lesser health-risk behavior; and healthcare requirements and costs have moved the working class, during their early to middle age, to plan directional outlines for their extended years in the workplace and senior post-retirement years in many developed and developing countries [3]. Unreliable health conditions and performance with lowered quality of life are concerning issues that adversely impact work sustainability. To minimize disability; planning for responsible behavior; and awareness about a sustainable lifestyle and the evolution of senescence is essential [4]. People selecting to work longer would require incorporating various resources to stay healthy and minimize disability. This would aid in continuing to work with desirable outcomes and avoiding health-related complications due to extended work schedule with the ever increasing pace, stress, and sophistication of workplace settings [5]. Therefore, the need to understand the concepts of work-life expectancy becomes very important to evaluate measures of work sustainability.

The work-life expectancy at a certain age is the average number of years that a person in a given cohort will spend either working or actively looking for work during the remainder of one’s life [6]. Markov assumption [7] for a work-life relates that the probability of a person under study would be active in the following year depends only on whether one was active or inactive in the present year. Even if the person has 20 previous years as an active member of the labor force; one’s chances of being inactive next year are the same as someone, who had been in and out of the labor force 10 times in the same period [8]. Therefore, the possibility of a person to remain in the labor force can be an outcome of a combination of necessities and abilities [9]. Given the state of current social and economic conditions, there is an increasing trend for workers to extend retirement plans and restructure post-retirement requirements. Specifically, this growing age group might need to make informed decisions for working longer than anticipated to accommodate already inflated financial goals [10]. Additional concern among older workers is a greater need for improvement in terms of training and health conditions comparing with the younger working population [11].

## 2. WORK-LIFE CONCERNS IN US AND SCOPE OF WORK SUSTAINABILITY

### 2.1. Implications of Work-Life Expectancy in US Context

Continuous population growth among the older group adults, apprehensions of financial planning and independence have become emerging gerontology issues. Increasing costs of healthcare services in United States have started to raise concerns in the working class to explore new approaches in an effort to sustain and meet the goals for prolonging workability, productivity ahead of time. Older Americans, the most rapidly growing age group, are the least physically active and generate the highest health care expenditures [12]. In next 20 years, United States will experience a major demographic shift as the largest birth cohort grows older [13]. Emerging adverse impacts of economic crisis with the inflation of healthcare costs for the elderly would not be sustainable, therefore significant policy development and their planned implementation in a timely manner would become necessary process for individuals and agencies to intervene adequately. Historical information about retirement rates could be used to trace out the long-term trends in retirement. The average retirement age is the youngest age at which at least half of population leave the labor force Historically, there was a progressive pattern towards earlier retirement in males (majority in US workforce) from 1910s, which has ceased by the late-1990s. [14]. Therefore, some programs that would need urgent amendments and reforms in recent future could be Medicare and Social Security. This could translate in reduced long-term care and health management of the elderly, technological support, involvement of geriatric population in work force, and development of viable strategies to pay for escalating medical care costs [15].

However all such programs and their applicability require reliability to ascertain the extent and involvement of various measures that could relate to the needs of the elderly for maintaining their healthy status as well as financial independence. Thus characterization of work sustainability indices in US context and evaluation of health promotion practices is needed. Applicable programs need to be developed for the working population to extend, improve their work-life. Such programs and approaches also in time-course could aid in furthering the understanding of variability in the level of severity of the problem and adequately generate implementation strategies.

### 2.2. Recommendations and Scope for Strategic Evaluation for Appropriating Intervention Planning

Longitudinal investigations are needed in US to evaluate health promotion practices, which could extend, improve and sustain work-life with trans-generational appropriations and assist the working population to adhere to intelligent programs. Present health practices and planning with the anticipation of prolonging the work-life for working population with aging concerns in US is not much studied yet. Such studies could potentially provide information about the choices and availability of appropriate health promotion practices; and how various working class populations can utilize such information for sustaining workability while transitioning through the older age. Standard survey questionnaires (namely biographical, occupational health surveys; economic expectancy questionnaire) could incorporate mental and physical assessments; and financial expectancy and independence projections congruently. Such tools could be useful to identify significant correlates of health promotion practices with indices of work-life sustainability [16,17]; some of the significant ones could include Employee Biographical Questionnaire (EBQ) and Occupational Health Surveillance Questionnaire (OHRQ). Such assessments should be implemented on various age cohorts of working population to expand the evaluation with age-dependent perspective. Identifying and evaluating such age-cohorts, data collection strategies and accuracy of evaluations would play highly sensitive role in such large demographical-longitudinal studies. Research questions directed towards the assessment of performance of the health advancing techniques that adult and senior-adult populations have utilized or would use to achieve would be very important to provide essential frame work of individual health. Investigation of resources, which people use to stay healthy and fit, would be able to justify the expectancy of responsible behavior and work-life longevity. Such study paradigms could lay the necessary groundwork to entitle a set of markers for health promotion practices. Results from such investigations, health and fitness practices and programs used by working population can also be evaluated on the basis of outcome of work-life sustainability and not only on achieving general healthy status.

Targeting various age-groups with psychometric evaluation designed to explain health promotion factors for sustaining work-life expectancy in aging population could provide opportunities to evaluate multigenerational variability as well. These factors could be used as determinants for further consolidating recommendations for various growing age groups for selection of available options to sustain workability. Ultimately, health practices could be used by younger working class as a tool to enhance working abilities by improving and maintaining health condition for projected workability while transitioning through their age, securing their potential to continue with their employment by optimizing their health status. These study outcomes could help employers as well for improving responsible behavior development among employees. It could essentially serve the employees as health promotion measures tailored to their needs, structuring the health incentives and regulating the insurance and healthcare costs thereby maintaining health status of their employees targeted towards sustaining workability [18]. Such broad spectrum studies could also be able to identify predictability of appropriate health promoting practices and vitality of translational planning strategies. Outcome of investigations to measure health indices for sustaining workability could potentially provide valuable information of successful methodologies used by various working classes in different age groups to develop and maintain their health status for conserving their optimal workability for a desirable time-frame in old age. Such investigations could further information regarding the choices, various sub-populations characterizations and recommendations of health profiling and modeling that could be used for developing intelligent health deigns and predictable planning approaches.
